# Can yoga effectively treat chronic back pain?

**Published:** 2024-03

**FEBRUARY 21, 2024 -** In the study, 10 women with and 11 without chronic low back pain underwent an 8‐session yoga program over 4 weeks, with the first session conducted in a clinic and the rest delivered with a tele‐approach. Women with chronic low back pain experienced a significant decrease in pain intensity, as assessed through a 10-point visual analog scale (an average pain of 6.80 at the start, dropped to 3.30 after the sessions) and through a spine-related measure called the flexion–relaxation phenomenon, which is often absent or disrupted in people with low back pain (5.12 at the start versus 9.49 after the sessions).

The findings suggest yoga can positively impact the neuromuscular response during trunk flexion and pain perception in individuals with chronic low back pain.

“It was interesting to show the role that yoga might play in the management of chronic back pain,” said corresponding author Prof. Alessandro de Sire, MD, of the University of Catanzaro “Magna Graecia” and University Hospital “Renato Dulbecco,” in Italy.

The authors noted that further research is warranted to assess yoga’s long‐term effects.

***Link to Study: https://onlinelibrary.wiley.com/doi/10.1002/jor.25790***

***Full citation:** “Impact of yoga asanas on flexion and relaxation phenomenon in women with chronic low back pain: Prophet model prospective study.” Nicola Marotta, Alessandro de Sire, Lorenzo Lippi, Lucrezia Moggio, Anna Tasselli, Marco Invernizzi, Antonio Ammendolia, Teresa Iona. J Orthop Res. Published Online: 21 February 2024 (DOI: 10.1002/jor.25790).*

*Copyright © 2019 The Cochrane Collaboration. Published by John Wiley & Sons, Ltd., reproduced with permission.*

